# NMDA Receptor-Dependent Synaptic Activity in Dorsal Motor Nucleus of Vagus Mediates the Enhancement of Gastric Motility by Stimulating ST36

**DOI:** 10.1155/2012/438460

**Published:** 2012-10-15

**Authors:** Xinyan Gao, Yongfa Qiao, Baohui Jia, Xianghong Jing, Bin Cheng, Lei Wen, Qiwen Tan, Yi Zhou, Bing Zhu, Haifa Qiao

**Affiliations:** ^1^Institute of Acupuncture and Moxibustion, China Academy of Chinese Medical Sciences, 16 Nanxiaojie Street, Dongzhimennei, Beijing 100700, China; ^2^Qingdao Haici Medical Group, 4 Renmin Road, Qingdao 266033, China; ^3^Guanganmen Hospital, China Academy of Chinese Medical Sciences, Beijing 100053, China; ^4^The Affiliated Hospital, Shandong University of Traditional Chinese Medicine, Jinan 250014, China; ^5^Department of Pharmacology, Southern Medical University, Guangzhou 510515, China; ^6^Department of Biomedical Sciences, Florida State University College of Medicine, Tallahassee, FL 32306, USA

## Abstract

Previous studies have demonstrated the efficacy of electroacupuncture at ST36 for patients with gastrointestinal motility disorders. While several lines of evidence suggest that the effect may involve vagal reflex, the precise molecular mechanism underlying this process still remains unclear. Here we report that the intragastric pressure increase induced by low frequency electric stimulation at ST36 was blocked by AP-5, an antagonist of N-methyl-D-aspartate receptors (NMDARs). Indeed, stimulating ST36 enhanced NMDAR-mediated, but not 2-amino-3-(5-methyl-3-oxo-1,2-oxazol-4-yl)propanoic-acid-(AMPA-) receptor-(AMPAR-) mediated synaptic transmission in gastric-projecting neurons of the dorsal motor nucleus of the vagus (DMV). We also identified that suppression of presynaptic **μ**-opioid receptors may contribute to upregulation of NMDAR-mediated synaptic transmission induced by electroacupuncture at ST36. Furthermore, we determined that the glutamate-receptor-2a-(NR2A-) containing NMDARs are essential for NMDAR-mediated enhancement of gastric motility caused by stimulating ST36. Taken together, our results reveal an important role of NMDA receptors in mediating enhancement of gastric motility induced by stimulating ST36.

## 1. Introduction

Gastric motility disorders are clinically characterized by impaired accommodation, gastroparesis, and dumping syndrome. A large number of studies has been conducted to explore the efficacy of somatic stimulation for the treatment of gastrointestinal motility disorders [1–5]. Reproducible results were generated in both clinical and research settings [6, 7], and several lines of evidence suggest that the gastric motility regulation induced by stimulating ST36 seems to be mediated via vagal reflex in the supraspinal pathway [5, 8–10]. However, how stimulating ST36 regulates gastric motility through relay nuclei and the molecular mechanism employed in this process still remain unclear. Addressing this question can provide valuable clues for the development of effective therapeutics against gastrointestinal motility disorders.

 Vagal motor innervation to the major portion of the gastrointestinal (GI) tract is provided by neurons in the dorsal motor nucleus of the vagus (DMV) [11, 12]. Nucleus of the solitary tract (NTS) neurons can potentially contribute input to the DMV and induce potent effects on vagus-mediated gastric function through excitatory glutamatergic and inhibitory GABAergic synaptic connections [13, 14]. Neuropharmacological studies have demonstrated changes in gastric function in response to localized application of gamma-aminobutyric acid (GABA) and glutamate (via GABAa and NMDA receptors) within the DMV [15–18]. 

 In this study, we directly examined the functional role of NMDAR-mediated synaptic transmission in mediating the upregulation of gastric motility by stimulating ST36. Our data reveal that electroacupuncture at ST36 upregulates gastric motility by specifically enhancing the glutamate-receptor-2a-(NR2A-) containing N-Methyl-D-aspartate-receptors-(NMDAR-) mediated synaptic transmission in gastric-projecting DMV neurons.

## 2. Materials and Methods

### 2.1. Animals and Surgical Preparation for *In Vivo* Experiments

Adult male Sprague Dawley (250–300 g) rats were purchased from the Institute of Laboratory Animalsciences, CAMS and PUMC (Beijing, China). In this study, all manipulations and procedures were carried out in accordance with The Guide for Care and Use of Laboratory Animals issued by USA National Institutes of Health and were approved by the Institutional Animal Care and Use Committee of China Academy of Chinese Medical Sciences. As described previously [19, 20], rats were housed (23 ± 1°C) in groups and maintained under a 12 hours light/dark cycle with food and water available *ad libitum*. The rats were fasted overnight with free access to water in *proxima luce*, and anesthetized with an intraperitoneal injection of urethane (1.0 g/kg, Sigma-Aldrich, St. Louis, USA). The left common carotid artery was cannulated with a polyethylene catheter filled with physiological saline containing heparin (200 IU/mL, LEO, Denmark) for recording of arterial pressure (AP) via a blood pressure transducer (TSD104A) and amplifier (MP150, DA100C, BIOPEC, Goleta, USA). The trachea was cannulated but not immobilized, to avoid respiratory tract congestion and a catheter was inserted into the left jugular vein for solution. A 2-mm-diameter polyurethane tube attached to a 1-cm-diameter latex balloon was inserted into the stomach through the mouth and esophagus. A syringe was attached to the cannula to inflate and deflate the balloon with water. The balloon was filled with 0.5–1.5 mL warm-water (37°C), which is equal to 80–150 mm H_2_O pressure, and the pressure was measured by a transducer connected to an amplifier through a thin polyethylene tube (1.5-mm in o.d.) and recorded by a multichannel data acquisition workstation (Micro1401-3, Cambridge Electronic Design, England). Offline data analysis was conducted with spike2 software. Semifasting gastric motor activity was recorded as a control for at least 1 hour before somatic stimulation. C57BL/6J or NR2A knockout mice (6–8 weeks) were purchased from Riken Bioresource Center, Japan and were treated with the protocol similar to the above. 

### 2.2. Microinjection in DMV

A glass micropipette (i.d.: 0.04 mm; o.d.: 0.12 mm; WPI, Sarasota, USA) with a tip diameter of ~30 *μ*m was stereotaxically placed at 0.1 to 0.6 mm rostral to calamus scriptorius (CS), 0.3 to 0.6 mm lateral from the midline, and 0.5 to 0.9 mm below the dorsal surface of the medulla. Microinjections of glutamate or GABA receptor (GABAR) antagonist were performed bilaterally via a Hamilton syringe (Mode 75) connected to the micropipette, with the movement of the meniscus monitored by a dissecting microscope. Injections were given in volumes of 20 nL over a period of 10–15 seconds. Somatic-stimulation-evoked responses were repeated 5 minutes after the DMV microinjection. The locations of microinjection were confirmed by histological verification.

### 2.3. Histological Verification of Injection Sites

The microinjection site in the brainstem was marked by pontamine sky blue. After fixing *in vivo* with 2% paraformaldehyde and 1% glutaraldehyde in 0.1 M PBS (pH 7.4), the brainstem was sectioned at 30 *μ*m, and the sections were stained with 0.3% neutral red. The marked microinjection site was located by microscopic examination. Only those data with histological and chemical confirmation were accepted.

### 2.4. Electroacupuncture

The stimulation electrode was placed at ST36, a hind limb point at which electroacupuncture or manual acupuncture enhances gastric motility [20]. Based on the descriptions in previous reports [21], the location is on the anterolateral side of the hind limb near the anterior crest of the tibia below the knee under the tibialis anterior muscle. This point was bilaterally stimulated with a 2-3 mA pulse of 0.5 ms duration at a frequency of 4 Hz for 30 seconds or 20 min by a pair of needle-electrodes inserted 3 mm depth into the skin. As a control, we selected CV12 which is located on the median line of the upper abdomen, 1.5 cm above the umbilicus, and could inhibit gastric motility [19]. The abdomen point was also inserted to a depth of 3 mm and stimulated with the same protocol. The electrical current for somatic stimulation was generated by a stimulator (SEN-7203, NIHON KOHDEN, Tokyo, Japan). For the recording of intragastric pressure *in vivo*, electroacupuncture was given at least 1 hour after stable basal recording or 10 min after drug administration. In brain slice experiments, we stimulated ST36 for 20 minutes in rats with retrograde labeling after anesthetization and then cut brain slices. 

### 2.5. Retrograde Labeling

Retrograde neuronal tracer 1,1′-dioctadecyl-3,3,3′,3′-tetramethylindocarbocyanine perchlorate (DiIC_18_(3); DiI) (Molecular Probes) was used to label gastric-projecting neurons of the DMV in 14-day-old male Sprague Dawley rats (Institute of Laboratory Animal Sciences, CAMS and PUMC, Beijing, China). As described previously [11, 22], after anesthetizing deeply with urethane and performing an abdominal laparotomy, DiI crystals were applied to one gastric region per rat (either the major curvature of the fundus or corpus or the antrum-pylorus). To confine the site of application, the crystals were embedded to the application site using a fast-settling epoxy resin that was allowed to harden for several minutes. After closing the laparotomy with 5/0 suture, the animals were placed in the chamber warmed under a radiant heat lamp until normal activity was restored. The animals were then returned to their home cages and allowed to recover for 10–15 days before brain slices were collected. 

### 2.6. Brain Slice Preparation

Thin brainstem slices were prepared from retrograde-labeled rats as described previously with several modifications [11, 22]. Briefly, the rat was sacrificed after being deeply anesthetized with urethane. The whole brain was then removed and placed in ice-cold artificial cerebrospinal fluid (ACSF) containing (mM): 124 NaCl, 3 KCl, 1.25 NaH_2_PO_4_, 1.3 MgSO_4_, 2 CaCl_2_, 26 NaHCO_3_, 10 glucose, bubbled with 95% O_2_/5% CO_2_, osmolality 300–310 mOsm. After removing the cerebellum, the brainstem was transected rostrally at the level of the pons and again at a point several millimeters caudal to the CS. A vibratome (VT1200S, Leica, German) was used to cut four to five coronal slices (250 *μ*m thickness) containing the DMV. The slices were incubated at 37°C for at least 45 minutes in oxygenated ACSF before use. 

### 2.7. Whole Cell Recording

A single slice was transferred to the recording chamber and kept in place with a slice anchor (Warner Instruments, Hamden, USA). The retrograde-labeled DMV neurons were identified under a Nikon E600 microscope (Nikon, Tokyo, Japan) equipped with tetramethylrhodamine isothiocyanate epifluorescence filters. Electrophysiological recordings were made under brightfield illumination after the identity of a labeled neuron was confirmed. The slice was continuously superfused with oxygenated ACSF (2 mL/min) at room temperature. Recording solution containing (in mM): 145 K-gluconate, 7.5 KCl, 9 NaCl, 1 MgSO_4_, 10 HEPES, 0.2 EGTA, 2 Na-ATP, 0.25 Na-GTP, adjusted to pH 7.4 with KOH, osmolality 290–300 mOsm, was used to back-fill recording electrodes (DC resistance: 5–7 MΩ). Currents were recorded with a MultiClamp700B amplifier (Molecular Devices) and filtered at 2 kHz with a lowpass filter, and data were digitized at 10 kHz and stored online using the pClamp10 software.

 For recording of mini-EPSC (mEPSC), the perfusion solution contained 30 *μ*M bicuculline and 1 *μ*M TTX, and the membrane was held at −60 mV. Data were analyzed with the Mini Analysis program (Synaptosoft, Leonia, USA).

For electrical stimulation-induced EPSCs, a concentric tungsten bipolar stimulating electrode (WPI, Sarasota, USA) was placed in the centralis or medialis subnuclei of the NTS. Single stimulus pulse (200 *μ*s, 10–500 *μ*A) or pairs of stimuli (200 *μ*s, 10–500 *μ*A, 100 ms interval) were applied every 20 seconds to evoke EPSCs. The above stimulation intensity is a range which can induce 50% of maximum AMPAR- or NMDAR-mediated EPSC. Series resistance ranged from 12 to 16 MΩ, and input resistance is 260–290 MΩ. The series and input resistances were monitored using voltage steps (5 mV, 50 ms) at 20-second intervals throughout the whole recording. If the membrane resistance changes more than 20% relative to an initial 3-minute period of recordings, the neuron will be rejected from the statistical analysis. Non-NMDAR- (AMPA/kainite-) and NMDAR-mediated EPSCs were recorded at a holding potential of −60 mV and +40 mV. 30 *μ*M bicuculline or 20 *μ*M 6,7-dinitroquinoxaline-2,3-dione (DNQX) was bath-applied to block GABAR or AMPA receptor (AMPAR) current. 5 mM QX314 was added to recording solution to prevent antidromically activated action potentials. Neurons were allowed to recover fully between additions of antagonists (minimum washout period of 10 minutes). Antagonists were superfused for at least 5 minutes. All chemicals or drugs are purchased from Sigma-Aldrich (St Louis, USA) if not stated otherwise.

### 2.8. Data Analysis

Data are shown as mean ± SEM. For significance evaluation, data sets with normal distribution were analyzed by paired or unpaired *t* test for two groups or one-way ANOVA followed by *q* test or Dunnett's test for more than two groups, and *P* < 0.05 was considered statistical significance.

## 3. Results

### 3.1. Electroacupuncture at ST36 Increases Gastric Motility through Activating DMV Neurons

**Figure 1:**
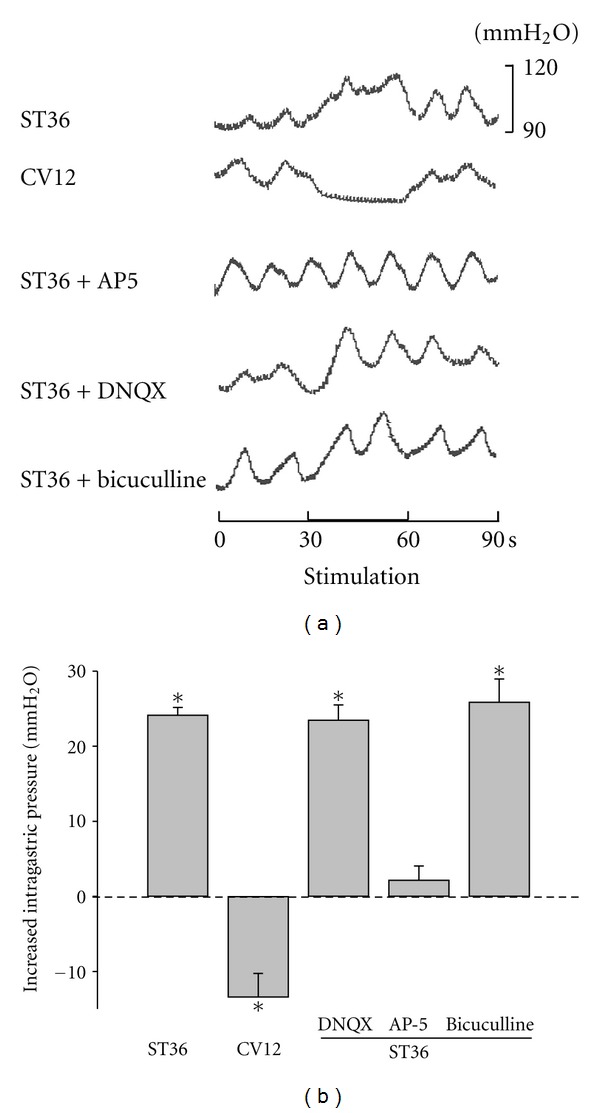
Low frequency stimulation at ST36 increases intragastric pressure. (a) Representative waves of intregastric pressure of rats induced by stimulating CV12 and ST36 with or without AP5, DNQX, or bicuculline. (b) Summarized data for the effect of low frequency stimulation at CV12 and ST36 with or without AP5, DNQX, or bicuculline on intragastric pressure. *P < 0.05, *n* = 9 for each group, one-way ANOVA followed by Dunnett's test.

 To determine whether electroacupuncture at ST36 may affect gastric motility in rats, we designed an experiment in which an electrical stimulation with 2-3 mA pulse of 0.2 ms duration at a frequency of 4 Hz was applied to ST36 or CV12. As shown in Figure 1(a), gastric pressure was dramatically increased by the low frequency stimulation at ST36. On the contrary, stimulating CV12 caused a marked reduction in gastric pressure. On average, gastric pressure was increased 24.15 ± 1.02 mm H_2_O (*P* < 0.05, *n* = 9) by stimulation at ST36, but decreased 13.43 ± 3.16 mm H_2_O (*P* < 0.05, *n* = 9) by stimulating CV12. Thus, these data suggested that low frequency stimulation at ST36 can regulate gastric motility in a location-specific manner. 

 To identify the role of glutamate or GABA receptors of DMV neurons in mediating the enhancement of gastric motility by electroacupuncture at ST36, we stereotaxically microinjected antagonists for either GABAa receptor bicuculline (2 nL, 30 *μ*M), or glutamate receptor including DNQX (2 nL, 20 *μ*M) for AMPARs and AP5 (2 nL, 50 *μ*M) for NMDARs into DMV. The increased gastric pressure induced by stimulating ST36 was significantly reduced by AP5 (2.18 ± 1.85 mm H_2_O, *P* < 0.05, *n* = 9), but not by bicuculline (25.89 ± 3.07 mm H_2_O, *P* > 0.05, *n* = 9) or DNQX (23.47 ± 2.05 mm H_2_O, *P* > 0.05, *n* = 9) (Figures 1(a) and 1(b)), suggesting that low frequency stimulation at ST36 increases gastric motility through activating NMDARs, rather than AMPARs or GABARs in DMV neurons.

### 3.2. Electroacupuncture at ST36 Enhances NMDAR-Mediated EPSCs in Gastric-Projecting DMV Neurons

**Figure 2:**
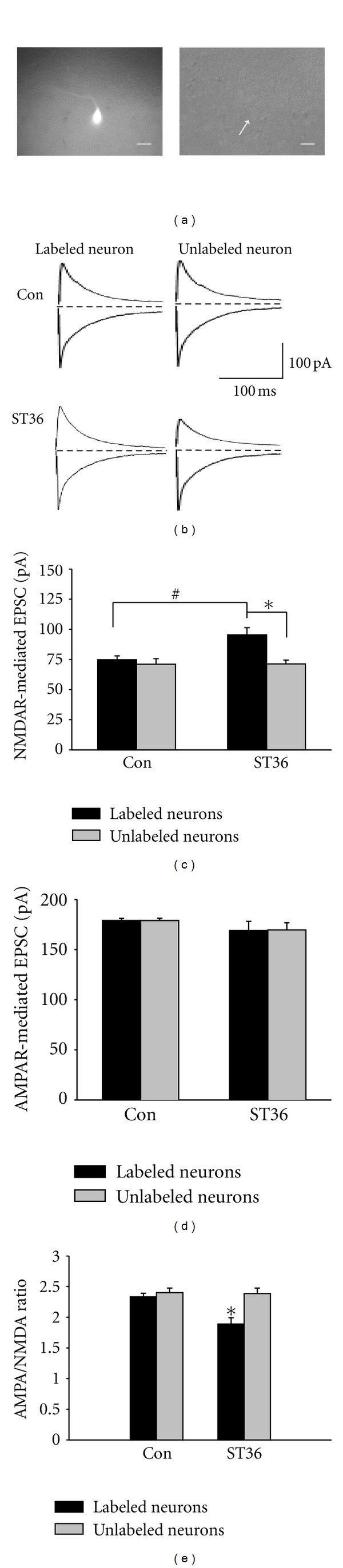
Low frequency stimulation at ST36 increases NMDAR-mediated EPSCs in gastric-projecting neurons. (a) Representative gastric-projecting neuron which is labeled with retrograde tracer. Left: under fluorescent light; right: under bright-field illumination. The arrows point to the same neuron. Calibration bar: 20 *μ*m. (b) Representative traces of labeled and unlabeled neurons in the brain slices from rats with and without low frequency stimulation at ST36. (c) NMDAR-mediated EPSCs are increased in labeled neurons from the stimulated rats. *P < 0.05, compared to unlabeled neurons from the stimulated rats, unpaired *t* test; ^#^ *P* < 0.05, compared to the labeled neurons from control rats (without stimulation), *n* = 12, unpaired *t* test. (d) The low frequency stimulation at ST36 does not change AMPA-receptor-(AMPAR-) mediated EPSCs in either labeled or unlabeled neurons, unpaired *t* test.

 To address whether electroacupuncture at ST36 specifically affects the NMDAR-mediated synaptic responses in gastric-projecting DMV neurons, we first used a retrograde tracing marker to label gastric-projecting DMV neurons. Similar to previous reports [11, 23, 24], a majority of labeled neurons were localized at the medial DMV and had small somas and few branches (Figure 2(a)). We then stimulated ST36 for 20 minutes in rats with retrograde labeling, and carried out whole cell recording in acute brainstem slices. In labeled DMV neurons, NMDAR-mediated EPSC in ST36 group was significantly larger than that in the control group without stimulation (91.49 ± 8.12 pA versus 68.50 ± 4.76 pA, *P* < 0.05, *n* = 12) (Figures 2(b) and 2(c)). By contrast, no significant difference was found in AMPAR-mediated EPSCs between ST36 and control groups in labeled DMV neurons (170.58 ± 11.74 pA versus 181.37 ± 4.19 pA, *P* > 0.05, *n* = 12) (Figures 2(b) and 2(d)). On the other hand, neither NMDAR nor AMPAR mediated EPSC in unlabeled DMV neurons had significant changes in either ST36 or control group (NMDAR-mediated EPSC: 69.17 ± 4.31 pA for stimulated versus 71.03 ± 5.89 pA for control, *P* > 0.05, *n* = 12; AMPAR-mediated EPSC: 182.05 ± 2.98 pA for stimulation versus 171.27 ± 8.42 pA for control, *P* > 0.05, *n* = 12) (Figures 2(b)–2(d)). As shown in Figure 2(e), AMPA/NMDA current ratio of labeled neurons in ST36 group decreased significantly compared to the unlabeled neurons (1.89 ± 0.10 for labeled neurons versus 2.39 ± 0.09 for unlabeled neurons, *P* < 0.05, *n* = 12); in control, no significant different between labeled or and unlabeled neurons was found (2.33 ± 0.06 for labeled neurons versus 2.40 ± 0.07 for unlabeled neurons). Thus, these results demonstrated that low frequency stimulation at ST36 selectively increased the NMDAR-mediated synaptic responses in gastric-projecting DMV neurons. 

### 3.3. Electroacupuncture at ST36 Increases NMDAR-Mediated Synaptic Transmission through Presynaptic Regulation

**Figure 3:**
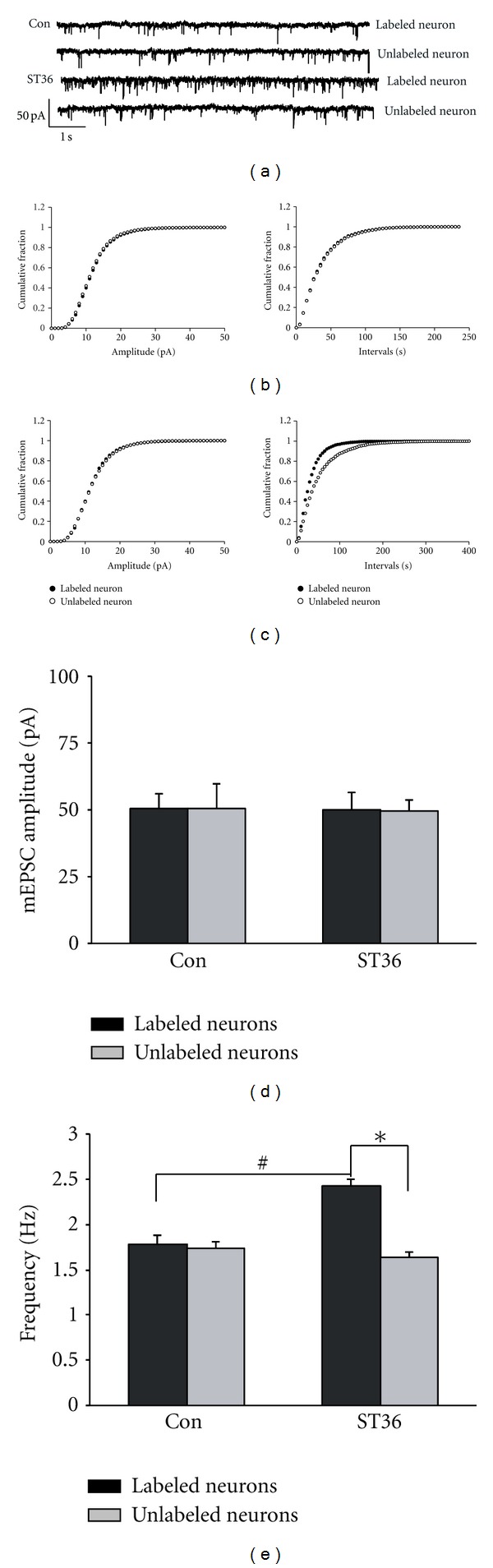
Low frequency stimulation at ST36 increases frequency of mEPSC. (a) Representative traces of mEPSC. (b) Cumulative plots of mEPSC amplitude and frequency of control. (c) Cumulative plots of mEPSC amplitude and frequency of low frequency stimulation at ST36. (d) Summarized amplitudes of mEPSC of labeled and unlabeled neuron in control and stimulated group. Unpaired *t* test shows no significant difference between labeled and unlabeled neurons in both groups. (e) Summarized frequencies of mEPSC of labeled and unlabeled neuron in control and stimulated rats. *P < 0.05, compared to the unlabeled neurons from the stimulated rats; ^#^ *P* < 0.05, compared to the labeled neurons from control; *n* = 8–11, unpaired *t* test.

 It is well documented that excitatory amino acid inputs from the NTS mediate vagal gastric motor excitation via NMDA and kainite/AMPA receptors in vagal motor neurons [25, 26]. Having identified that low frequency stimulation at ST36 increased NMDAR-mediated EPSCs between NTS and DMV EPSC, we went on to identify the pre- or postsynaptic mechanism responsible for the impact of electroacupuncture at ST36 on synaptic transmission. At first, mEPSCs were recorded in acute brainstem slices from rats subjected to ST36 stimulation. Although mEPSC amplitude did not differ between retrogradely labeled and unlabeled DMV neurons (51.41 ± 8.02 pA versus 50.07 ± 5.12 pA, *P* > 0.05, *n* = 11) (Figures 3(a)–3(d)), the frequency of mEPSC was significantly greater in labeled neurons (labeled neurons: 2.43 ± 0.07 Hz versus unlabeled neurons: 1.64 ± 0.05 Hz, *P* < 0.05, *n* = 11) (Figures 3(a)–3(c), and 3(e)). In control animals without somatic stimulation, we did not observe any significant difference in either mEPSC amplitude or frequency between labeled and unlabeled DMV neurons (amplitude: 51.38 ± 6.29 pA and frequency: 1.78 ± 0.10 Hz for labeled neurons versus 51.67 ± 9.88 pA and 1.74 ± 0.07 Hz for unlabeled neurons, *P* > 0.05, *n* = 8) (Figures 3(a), 3(b), 3(d), and 3(e)). 

**Figure 4:**
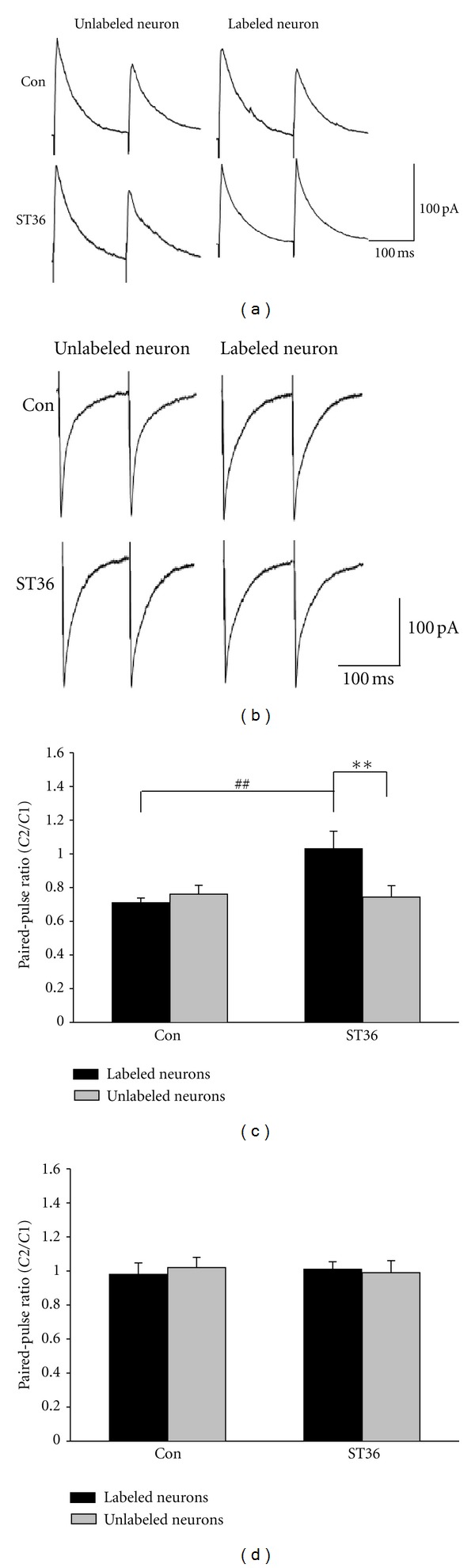
Low frequency stimulation at ST36 decreases paired-pulse ratio (PPR) of NMDAR-mediated EPSC. (a), (b) Representative traces of NMDAR- or AMPAR-mediated EPSC induced by paired-pulse stimulation in NTS. (c) ST36 stimulation decreases PPR of NMDAR-mediated EPSC in labeled DMV neurons. **P < 0.01, compared to the unlabeled neurons from the stimulated group; ^##^ *P* < 0.01, compared to the labeled neurons from control; *n* = 10, unpaired *t* test. (d) ST36 stimulation fails to cause significant change of PPR of AMPAR-mediated EPSC in labeled and unlabeled neurons in both groups, unpaired *t* test.

 In addition, we assessed the site of action by measuring the ratio of the amplitudes of two postsynaptic currents in DMV neurons using a paired-pulse protocol. By delivering paired-pulse (10 Hz) stimulation to presynaptic NTS, we consistently observed a substantial change in paired-pulse ratio (PPR) of NMDAR-mediated currents in labeled and unlabeled neurons. As shown in Figures 4(a) and 4(c), the PPR of NMDAR-mediated EPSCs recorded from the retrogradely labeled DMV neurons was significantly larger in ST36 group than that in control (1.03 ± 0.11 versus 0.71 + 0.02; *P* < 0.01, *n* = 10). In fact, there was a paired-pulse facilitation in labeled neurons compared to paired-pulse depression in unlabeled neurons. For the ST36 group, there was also a significant difference between PPR of NMDAR-mediated EPSCs in labeled and unlabeled neurons (1.03 ± 0.11 for labeled neurons versus 0.74 ± 0.07 for unlabeled neurons, *P* < 0.01, *n* = 10) (Figures 4(a) and 4(c)). By contrast, there was little changes in PPR of AMPAR-mediated EPSCs after ST36 stimulation, as the PPR recorded from labeled neuron in stimulated rats was not different with that either recorded from labeled neurons in the control group or unlabeled neurons in ST36 group (0.66 ± 0.04 for labeled neurons in ST36 group versus 0.72 ± 0.07 for labeled neurons in control; and 0.69 ± 0.07 for unlabeled neurons: *P* > 0.05, *n* = 10) (Figures 4(a)–4(d)). Given that a PPR change is indicative of a presynaptic site of action [27], these data suggested that stimulating ST36 increased NMDAR-mediated synaptic transmission via a presynaptic mechanism. 

### 3.4. Electroacupuncture at ST36 Inhibits Presynaptic *μ*-Opioid Receptors

**Figure 5:**
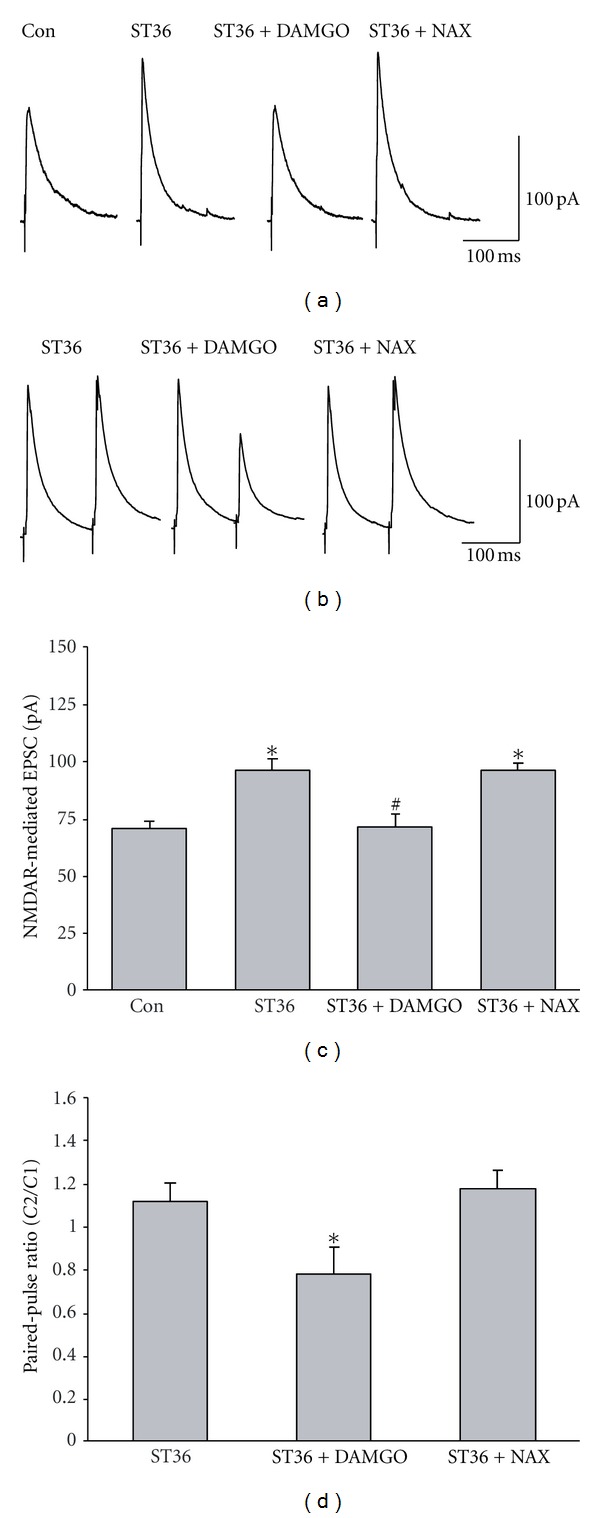
*μ*-opioid receptor agonist DAMGO abolishes the increase in NMDAR-mediated EPSC induced by low frequency stimulation at ST36. (a) Representative traces of NMDAR-mediated EPSC. (b) The increase of NMDAR-mediated EPSC in labeled DMV neurons induced by ST36 stimulation is reduced by bath perfusion of *μ*-opioid receptor agonist DAMGO and reversed by a specific *μ*-opioid receptor antagonist naloxonazine. *P < 0.05, compared to control; ^#^ *P* < 0.05, compared to the stimulated rats, *n* = 12–14, one-way ANOVA followed by *q* test. (c) Representative traces of NMDAR-mediated EPSC induced by paired-pulse. (d) The PPR decreased by ST36 stimulation is increased by DAMGO. *P < 0.05, *n* = 7-8, compared to ST36 stimulation and ST36 stimulation with naloxonazine, one-way ANOVA followed by *q* test.

Previous evidence indicates that increasing activity of the presynaptic *μ*-opioid receptors attenuates the excitatory synaptic transmission from the NTS to GI-projecting DMV neurons [28]. Here we set out to detect whether stimulating ST36 can affect presynaptic *μ*-opioid receptors and hence increase NMDAR-mediated synaptic transmission. As shown in Figures 5(a) and 5(c), the NMDAR-mediated EPSCs in labeled neurons were significantly reduced by perfusion of the brain slice with a competitive agonist of *μ*-opioid receptors, D-Ala^2^, N-MePhe^4^, Gly^5^-ol-enkephalin (DAMGO) (95.73 ± 4.89 pA versus 70.34 ± 7.22 pA. *P* < 0.05, *n* = 12–14), and the reduction in NMDAR-mediated EPSCs by DAMGO was reversed by 0.2 *μ*m naloxonazine, a selective *μ*-opioid receptor antagonist (93.39 ± 3.97 pA, *n* = 14). In addition, we found that the enhanced PPR by ST36 stimulation was abolished by DAMGO (stimulation: 1.12 ± 0.10 versus stimulation + DAMGO: 0.78 ± 0.13. *P* < 0.05, *n* = 7-8), and reversed by naloxonazine (1.18 ± 0.09, *P* < 0.05, *n* = 7) (Figures 5(b) and 5(d)). Taken together, these data suggested that low frequency stimulation at ST36 inhibits the presynaptic *μ*-opioid receptor. 

### 3.5. Electroacupuncture at ST36 Increases NR2A-Containing NMDAR-Mediated Synaptic Transmission of Gastric-Projecting DMV Neurons

**Figure 6:**
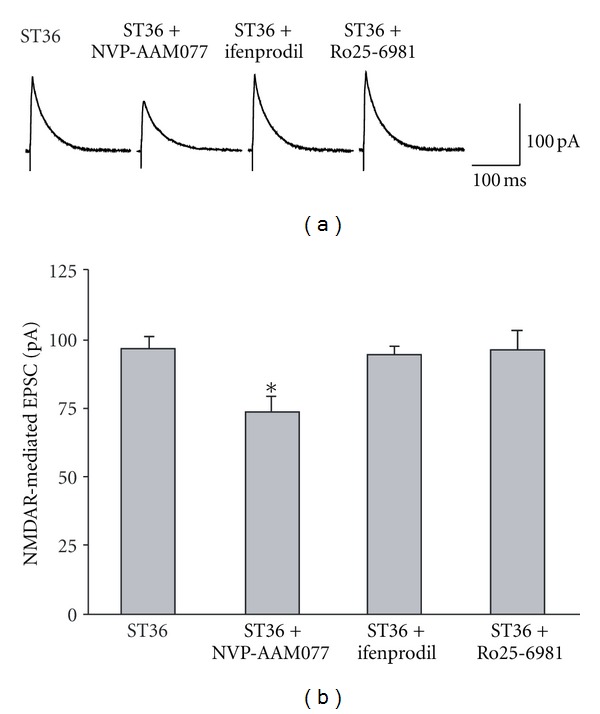
The increased NMDAR-mediated EPSC by low frequency stimulation at ST36 is diminished by NR2A antagonist NVP-AAM077. (a) Representative trace of NMDA-mediated EPSC with ST36 stimulation and different drugs. (b) The increased NMDAR-mediated EPSC by ST36 stimulation is diminished by NR2A antagonist NVP-AAM077, but not by NR2B antagonist ifenprodil and Ro25-6981. *P < 0.05,  *n* = 7–12, one-way ANOVA followed by Dunnett's test.

To further characterize the role of specific subunits of NMDAR in mediating regulation of gastric motility by stimulating ST36, we applied selective antagonists which block either NR2A or NR2B containing NMDARs during whole-cell recording in the retrograde labeling neurons. Figures 6(a) and 6(b) show that in the presence of a NR2A-specific antagonist, ((R)-((S)-1-(4-bromophenyl)-ethylamino)-(2,3-dioxo-1,2,3,4-tetrahydroquinoxalin-5-yl)-methyl)-phosphonic acid (NVP-AAM077) (0.4 *μ*M), the facilitation of NMDAR-mediated EPSCs induced by ST36 stimulation in labeled neurons was abolished completely (stimulation: 95.73 ± 4.89 pA, *n* = 12; NVP-AAM077: 70.47 ± 7.87 pA, *n* = 8; *P* < 0.05). Conversely, no significant difference was observed between pre- and postapplication of 3 *μ*M ifenprodil, a NR2B antagonist [29] (92.59 ± 4.07 pA, *n* = 7). Another NR2B-specific antagonist Ro25-6981 (0.5 *μ*M) [30] also did not cause a significant change in evoked NMDAR-mediated EPSCs (95.39 ± 7.94 pA, *n* = 7), suggesting that NR2A-containing NMDARs are essential for NMDAR-mediated increase of gastric motility by stimulating ST36.

### 3.6. NVP-AAM077 Microinjection Diminishes the Enhancement of Gastric Motility Induced by Electroacupuncture at ST36 in Anesthetized Rats and Transgenic Mice

**Figure 7:**
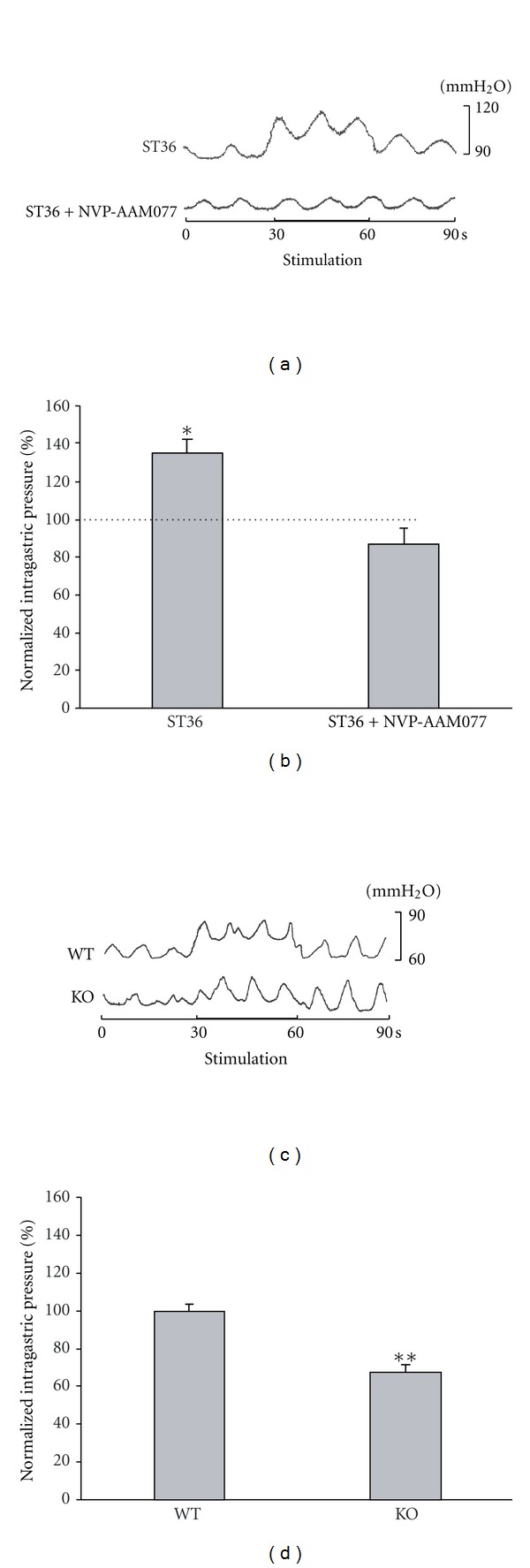
NVP-AAM077 microinjection of DMV reduced intragastric pressure enhanced by low frequency stimulation at ST36. (a) Representative waves of intragastric pressure under different conditions in anesthetized rats. (b) NVP-AAM077 abolishes the increase of intragastric pressure caused by ST36 stimulation. *P < 0.05, *n* = 5, one-way ANOVA followed by *q* test. Dotted line represents the control. (c) Representative waves of gastric motility of NR2A knockout mouse and its littermate wildtype. (d) ST36 stimulation increases intragastric pressure in wildtype mice but not in NR2A knockout mice. **P < 0.01, *n* = 5, unpaired *t* test.

To examine whether these findings for the role of NR2A-containing NMDARs observed *in vitro* could be reproduced *in vivo*, NVP-AAM077 (2 nL, 0.4 *μ*M) was microinjected into DMV before ST36 stimulation in anesthetized rats. As shown in Figures 7(a) and 7(b), the increase of the intragastric pressure induced by stimulating ST36 was diminished significantly after microinjection of NVP-AAM077 (135.15 ± 7.06% versus 87.04 ± 8.32%, *P* < 0.05, *n* = 5). To further verify a specific role of NR2A in mediating the regulation of gastric motility by stimulating ST36, we performed the same stimulation protocol in both wild-type and NR2A knockout mice. As shown in Figures 7(c) and 7(d), low frequency stimulation at ST increased gastric motility in the wild-type littermates, but not in the NR2A knockout mice (100.00 ± 3.96%, versus 67.97 ± 4.13%, *P* < 0.01, *n* = 5), providing additional evidence that NR2A-containing NMDARs of DMV neurons are required for the increase of gastric motility induced by stimulating ST36. 

## 4. Discussion

In the present study, we discovered that NMDARs of gastric-projecting DMV neurons play a critical role in mediating the enhancement of gastric motility induced by electroacupuncture at ST36 in anesthetized rats. Stimulating ST36 enhances NMDAR-mediated synaptic transmission through inhibiting presynaptic *μ*-opioid receptors. We also determined that the enhancement of NR2A-containing NMDAR-mediated synaptic transmission between NTS and gastric-projecting DMV neurons is an absolute requirement for this potent regulation of gastric motility. Therefore, our study unveiled a novel molecular mechanism by which stimulating ST36 upregulates gastric motility via the vagal pathway.

Somatovisceral reflexes responsible for regulation of visceral organs are strongly associated with the effects of acupuncture. Application of acupuncture to the abdominal region inhibits gastric motility in anesthetized rats via a spinal reflex that activates sympathetic efferent nerve fibers, while stimulating a limb increases gastric motility via a supra-spinal reflex that activates vagal nerve fibers [19, 31, 32]. Here, we found that low frequency stimulation at ST36 can significantly increase gastric motility, but stimulating CV12 decreases gastric motility significantly. Our results are consistent with previous reports and provide strong support for the location specificity of somatic stimulation in regulating gastric motility.

 It is well documented that somatic stimulation upregulates gastric motility through the vagal reflex pathway [5, 19]. However, little is known regarding the anatomic circuit and functional changes underlying the somatic stimulation induced regulation of gastric motility. In the vagal reflex pathway, DMV plays a pivotal role in controlling the motility of the GI tract, as it sends efferent projections to the GI tract. NTS neurons also regulate gastric motility by providing direct inhibitory and excitatory inputs to preganglionic parasympathetic neurons in the DMV. Based on previous studies in brainstem slice preparations, endogenously occurring glutamate and GABA are the major neurotransmitters controlling the excitability of DMV motor neurons [24], while their inhibitory and excitatory effects on the excitability of DMV neurons are mediated directly via activation of postsynaptic GABAa receptors and both NMDA- and non-NMDA-type glutamatergic receptors [26, 33, 34]. Our finding that the increase in intragastric pressure induced by low frequency stimulation at ST36 was diminished by NMDAR antagonist AP5 indicates that low frequency stimulation at ST36 upregulates gastric motility through the excitatory synaptic transmission between NTS and DMV, especially through NMDARs rather than non-NMDARs of DMV neurons. 

The distinct distribution and morphology are characteristics of a subpopulation of DMV neurons that project to the stomach [13]. Our finding that stimulating ST36 enhances NMDAR-mediated EPSCs specifically in the retrogradely labeled neurons suggests that NMDARs of gastric-projecting DMV neurons mediate the regulation of gastric motility by somatic stimulation. We also determined that the ST36 stimulation induced enhancement of NMDAR-mediated synaptic transmission in DMV neurons is through presynaptic regulation, and propose inhibition of presynaptic *μ*-opioid receptors as one of the potential mechanisms. This hypothesis is supported by previous observations that opioid peptides attenuate excitatory synaptic transmission to gastric-projecting DMV neurons via interactions with presynaptic *μ*-opioid receptors [27]. In addition, opioid pathway may contribute to long-lasting effects of acupuncture on gastric motility [35]. The current result that *μ*-opioid receptor agonist DAMGO inhibited EPSCs of the gastric-projecting DMV neurons also suggests that increasing NMDAR-mediated synaptic transmission may be attributed to the inhibition of presynaptic *μ*-opioid receptors. However, the data that low frequency stimulation at ST36 did not change AMPAR-mediated synaptic transmission implies that other mechanisms might be involved and also need to be clarified in the future.

In the present work, we demonstrate that NMDAR-mediated excitation of DMV neurons evoked by ST36 stimulation is primarily mediated by NR1/NR2A receptors, as NR2A preferential antagonist *in vitro* decreases NMDAR-mediated synaptic response and *in vivo* abolishes the increase of gastric motility induced by low frequency somatic stimulation. The observed involvement of NR2A receptors in NMDAR-mediated upregulation of gastric motility by low frequency stimulation at ST36 is in agreement with previous reports that, in the adult brainstem, NR2A is predominantly expressed among the four NR2 subunits [36, 37] and the functional change in NMDAR properties is correlated with an increase in the NR2A subunit ratio [37, 38]. 

## 5. Conclusions

To sum, our study establishes that low frequency somatic stimulation at ST36 enhances NMDAR-mediated synaptic transmission via suppressing presynaptic *μ*-opioid receptors, and in turn increases NR2A-containing NMDAR-mediated synaptic transmission in gastric-projecting DMV neurons. While future studies are needed to clarify how low frequency stimulation at ST36 inhibits presynaptic *μ*-opioid receptors and thus increases NMDAR-mediated synaptic transmission, our data provide an important insight into the mechanism for ST36 stimulation enhanced gastric motility. In the future, this finding may also help to develop treatment strategy for gastric motility disorders by an NR2A-containing NMDAR-based activation approach.
